# The Identification, Characterization, and Functional Analysis of the Sugar Transporter Gene Family of the Rice False Smut Pathogen, *Villosiclava virens*

**DOI:** 10.3390/ijms25010600

**Published:** 2024-01-02

**Authors:** Huimin Qin, Weixiao Yin, Chaoxi Luo, Lianmeng Liu

**Affiliations:** 1 State Key Laboratory of Rice Biology and Breeding, China National Rice Research Institute, Hangzhou 311400, China; qhm2916@163.com; 2 College of Plant Science and Technology, Huazhong Agricultural University, Wuhan 430070, China; wxyin@mail.hzau.edu.cn

**Keywords:** false smut, *Villosiclava virens*, *Ustilaginoidea virens*, sugar transporter, STP, expression pattern, pathogenicity

## Abstract

False smut, caused by *Villosiclava virens*, is becoming increasingly serious in modern rice production systems, leading to yield losses and quality declines. Successful infection requires efficient acquisition of sucrose, abundant in rice panicles, as well as other sugars. Sugar transporters (STPs) may play an important role in this process. STPs belong to a major facilitator superfamily, which consists of large multigenic families necessary to partition sugars between fungal pathogens and their hosts. This study identified and characterized the STP family of *V. viren*, and further analyzed their gene functions to uncover their roles in interactions with rice. Through genome-wide and systematic bioinformatics analyses, 35 STPs were identified from V.virens and named from *VvSTP1* to *VvSTP35*. Transmembrane domains, gene structures, and conserved motifs of VvSTPs have been identified and characterized through the bioinformatic analysis. In addition, a phylogenetic analysis revealed relationship between VvSTPs and STPs from the other three reference fungi. According to a qRT-PCR and RNA-sequencing analysis, *VvSTP* expression responded differently to different sole carbon sources and H_2_O_2_ treatments, and changed during the pathogenic process, suggesting that these proteins are involved in interactions with rice and potentially functional in pathogenesis. In total, 12 representative VvSTPs were knocked out through genetic recombination in order to analyze their roles in pathogenicity of *V. virens*. The knock-out mutants of VvSTPs showed little difference in mycelia growth and conidiation, indicating a single gene in this family cannot influence vegetative growth of *V. virens*. It is clear, however, that these mutants result in a change in infection efficiency in a different way, indicating that VvSTPs play an important role in the pathogenicity of virens. This study is expected to contribute to a better understanding of how host-derived sugars contribute to *V. virens* pathogenicity.

## 1. Introduction

In animals, plants, and fungi, sugar transporters (STPs) are large multigene families that play a key role in transmembrane transport and distribution of sugars, which is essential to sugar partitioning between fungal pathogens and their hosts [1,2,3]. The sugar transporters belong to a major facilitator superfamily, which has 12 transmembrane domains connected by hydrophilic loops. They can be divided into two distinct classes, monosaccharide transporters (MSTs) and sucrose transporters (SUTs), based on the sugar classes they carry [4,5,6].

A number of fungal sugar transporters have been demonstrated to be involved in plant–fungus interactions, including mutualistic and pathogenic interactions. Most of these fungal sugar transporters are MSTs. These transporters are specific for monosaccharides and provide fungi with a monosaccharide carbon source mostly derived from extracellular sucrose hydrolysis by fungal- and/or plant-derived cell wall invertases [7,8,9,10,11]. The first MST to be confirmed for its involvement in pathogenic fungal–plant interactions was *UfHXT1*, which showed high affinity for glucose in *Uromyces fabae*, the pathogen responsible for faba-bean rust [12]. Later, *GpMST1* from *Geosiphon pyriformis* and *TBHXT1* from *Tuber borchii* were proven to facilitate the uptake of carbohydrates by mycorrhizal fungi [13,14]. The hydrolysis of extracellular sucrose results in an increase in apoplastic glucose concentrations, which can be sensed by plants and trigger their defense mechanisms [15,16,17,18]. Therefore, it might be more advantageous for plant pathogenic fungi to feed directly on sucrose instead of invertase-derived glucose [7]. A novel and plasma-membrane-localized sucrose-specific transporter, *Srt1*, has been identified as a fungal virulence factor of *Ustilago maydis*. Due to its unusually high substrate affinity, this transporter effectively competes for sucrose against its plant-derived counterparts. Thus, the pathogens are able to utilize sucrose at the plant/fungal interface directly without generating extracellular monosaccharides that are known to elicit plant immune responses [7]. According to these findings, both fungal SUTs and MSTs have an important role to play in plant–fungal interactions.

Rice false smut caused by *Villosiclava virens* (anamorph: *Ustilaginoidea virens*) has a long epidemic history in rice planting and was recorded in *Compendium of Materia Medica*, an ancient Chinese book written from 1552 to 1578 [19,20,21]. It has been reported that the disease not only causes yield loss, but it might also cause the grain to be polluted with dozens of mycotoxins that are hazardous to consumers, thereby arousing growing public concern [22,23]. As a result of the proliferation of high-yielding, long-growing hybrid rice varieties in recent years, particularly intersubspecific hybrids of indica–japonica, extensive planting, excessive water use, and excessive chemical fertilizer use, rice false smut has become a major issue from a minor and neglected rice disease [24,25,26], and is estimated to occur in one-third of the rice cultivation areas in China, which poses a substantial threat to rice production [27,28], as well as food safety.

*V. virens* is most likely to obtain its carbon nutrient from rice in the form of sugars. Sugar absorption from rice could be a key process in its pathogenic mechanism. Among all rice diseases, false smut balls, which are several times larger than mature rice seeds, are the most obvious distinguishing feature of the pathogen. The rice false smut fungus must obtain sufficient energy from rice in an efficient manner in order to form such large smut balls. According to our previous studies, *V. virens* hijacks rice nutrients by blocking and mimicking rice ovary fertilization [29]. The majority of photosynthetic products produced with fixed solar energy are transported from source leaves to sink ovaries as sugars, of which sucrose is the most common form and sugars such as raffinose, stachyose, and polyols are also transported. For transport between source and sink organs, sugar gradients are an important driving force [2,30,31]. Part of the sucrose transported from source organs could be hydrolyzed by plant or fungal invertases into glucose and fructose in the ovary. Sucrose and other sugars are abundant in rice spikelets and could be taken up to support growth of *V. virens*. Therefore, we hypothesized that sugar transporters in the STP family could play an important role in pathogenesis of *V. virens*.

Currently, research of *V. virens* is still in the start-up phase, and knowledge of its genes and proteins is very limited. *V. virens* genome sequencing has already opened a door for molecular biology studies [32]; however, information on its STP gene family is still insufficient and needs intensive further research.

The VvSTP gene family was identified in this study by searching the published genome, and its members were analyzed through genome-wide systematic characterization. Quantitative RT-PCR (qRT-PCR) was used to investigate the response of VvSTP genes to different substrates, and RNA sequencing was used to confirm the presence of involved VvSTP genes during the infection process. Lastly, mutants of these genes were constructed and their roles in pathogenesis were revealed by studying these mutants. As a result of this study, we gained a greater understanding of the STP gene family of *V. virens*, and we obtained preliminary information regarding their transcriptional regulation mechanisms, which provides a useful starting point for further investigation of their potential roles in pathogenesis.

## 2. Results

### 2.1. Identification of the STP Family in V. virens

After searching in the protein set of *V. virens*, 73 sequences were screened using the HEMMER program at the threshold of an E-value < 0.01. Then, the sequences were submitted to a CD-search in NCBI, and sequences without a sugar_tr superfamily domain were manually removed according to the results. Finally, 35 sequences were identified as *VvSTP1*-*VvSTP35* (Table 1).

In the genome database of *V. virens* UV-8b (JHTR00000000.1), 35 VvSTPs are spread on all seven chromosomes. The predicted proteins ranged in amino acid length from 497 to 777, and their MWs ranged from 54.12 kDa to 86.56 kDa (Table 1). VvSTPs have a PI ranging from 5.13 to 9.34, with 20 of 35 having a PI greater than 8 (Table 1). All VvSTPs were predicted to contain 12 transmembrane domains. All were also predicted to be localized to the plasma membrane (Table 1).

### 2.2. Phylogenetic Relationship, Gene Structure, Conserved Motifs of STP Genes of V. virens

**Figure 1:**
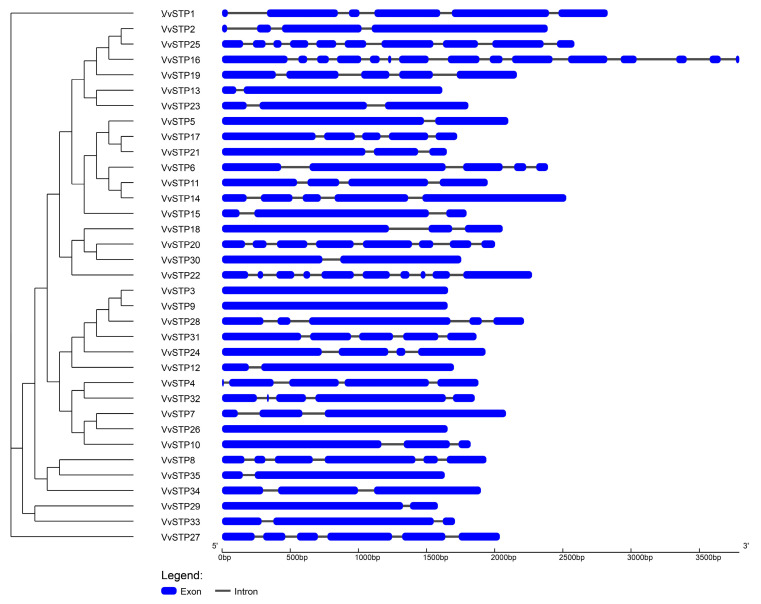
Gene structure of 35 VvSTPs. Scales of the exons are represented by blue boxes. Gray lines connecting two exons represent the introns. Left panel shows phylogenetic tree built with all 35 VvSTPs through maximum-likelihood (ML) method.

The VvSTPs were divided into groups based on the results of phylogenetic analyses within the gene family, as illustrated in Figure 1. *VvSTP1* and *VvSTP27* were distantly related to other VvSTPs and to each other. At the fourth node, most VvSTPs were clustered into one group except *VvSTP1*, *VvSTP8*, *VvSTP27*, *VvSTP29*, *VvSTP33*, *VvSTP34*, and *VvSTP35*.

The number of introns in VvSTPs ranged from 0 to 14. Three VvSTPs were intronless, including *VvSTP3*, *VvSTP9*, and *VvSTP26*. In contrast, *VvSTP16* exhibits an intron count as high as 14, followed by *VvSTP22* and *VvSTP25*, each of which contain 9 introns. The length of the introns of VvSTPs ranged from 38 to 290, while the length of the extrons ranged from 12 to 1659 (Figure 1).

**Figure 2:**
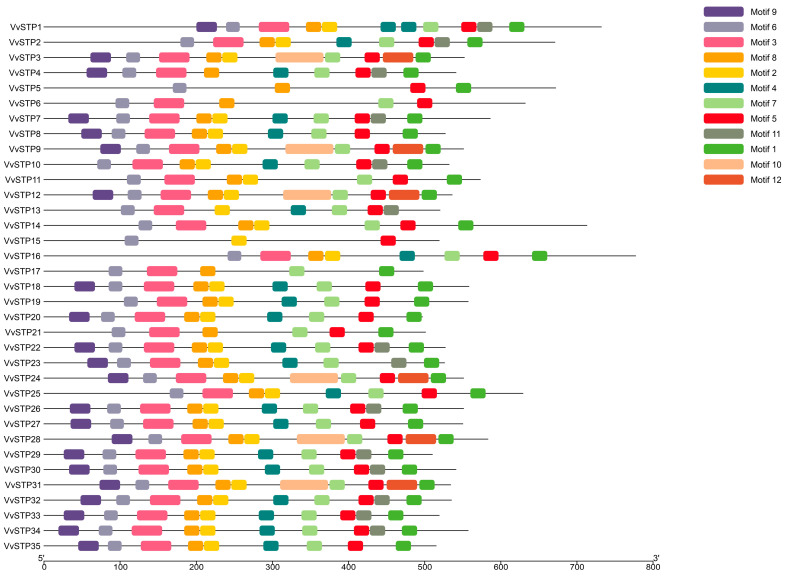
Distribution of conserved motifs of all 35 *VvSTP*s identified by MEME with the amino acid sequences. Conserved motifs are length-proportional and presented by boxes of different colors among the none-conserved sequences represented by gray lines.

MEME Suite detected 12 conserved motifs of STP in VvSTPs using AA sequences. There were 19 to 64 amino acid residues in the conserved motifs, and all VvSTPs contained at least 3 of the conserved motifs (*VvSTP15*), and at most 11 motifs (*VvSTP1*). There were 10 conserved motifs in 14 VvSTPs, the most common motif number, followed by 9 VvSTPs with 9 motifs. There was a higher degree of conservation of motif 5 and 6 in VvSTPs, presenting in all 35 VvSTPs (Figure 2).

The duplication of VvSTP genes was analyzed using MCScanX. As a result of the study, VvSTPs were distributed on seven chromosomes of the genome of *V. virens* strain UV-8b. No whole genome duplications (WGDs) were detected, but four pairs of tandem duplication genes were identified, including *VvSTP31* and *VvSTP32*, *VvSTP27* and *VvSTP28*, *VvSTP5* and *VvSTP6*, and *VvSTP1* and *VvSTP2*.

**Figure 3:**
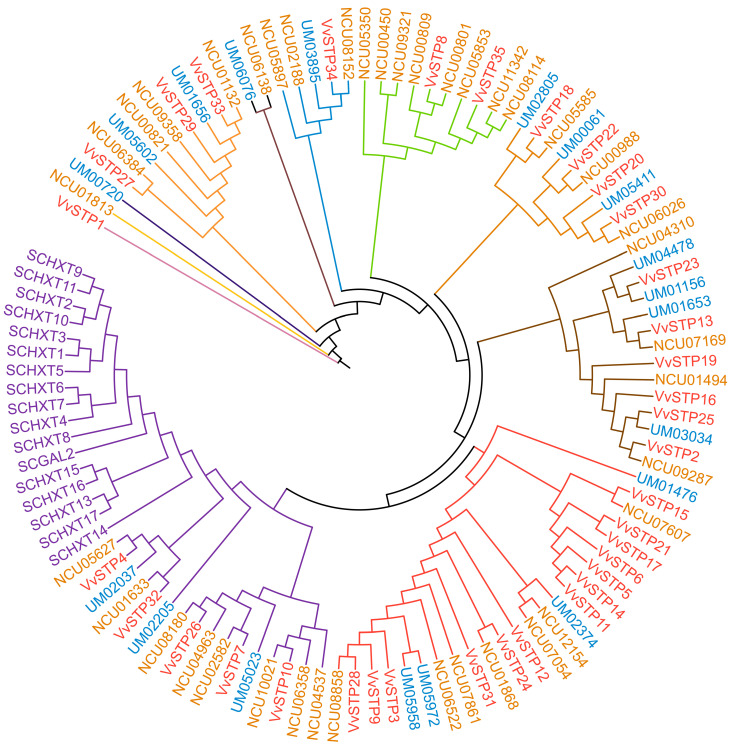
Phylogenetic relation of VvSTPs with three other fungal STP proteins. Labels of STP proteins from *Villosiclava virens*, *Saccharomyces cerevisiae*, *Neurospora crassa*, and *Ustilago maydis* are shown in different colors, red, purple, brown, and blue, respectively. Branch of different clades is also shown in different colors.

All STPs for the analysis were grouped into 11 clades, which were labeled with different colors, by the tenth node of the phylogenetic tree (Figure 3). Since *S. cerevisiae* is a yeast, it is not surprising that all of its STPs are clustered in a clade without another STP gene. STPs from all three filamentous fungi interlaced. However, most of the VvSTPs belonged to the clade whose branch was colored red, and shared a father clade with STPs of *S. cerevisiae*, which indicates that most VvSTPs are more closely related to those of *S. cerevisiae* than STPs from two other filamentous fungi. Compared with other VvSTPs and the STPs of the other two reference fungi, *VvSTP1* showed distant similarities, which suggests that it may be a unique STP and differ from other known fungal STPs. Except for *VvSTP1*, *VvSTP27* and *VvSTP29* are also more distantly related to other VvSTPs. *VvSTP8* and *VvSTP35* showed a closer relationship with STPs from *N. crassa*, such as NCU09321, than with the majority of VvSTPs. Several STP genes from *N. crassa* and *U. maydis*, such as NCU07607 and UM03274, were grouped with VvSTP genes.

### 2.3. Expression Pattern of VvSTPs on Different Sole Carbon Sources

**Figure 4:**
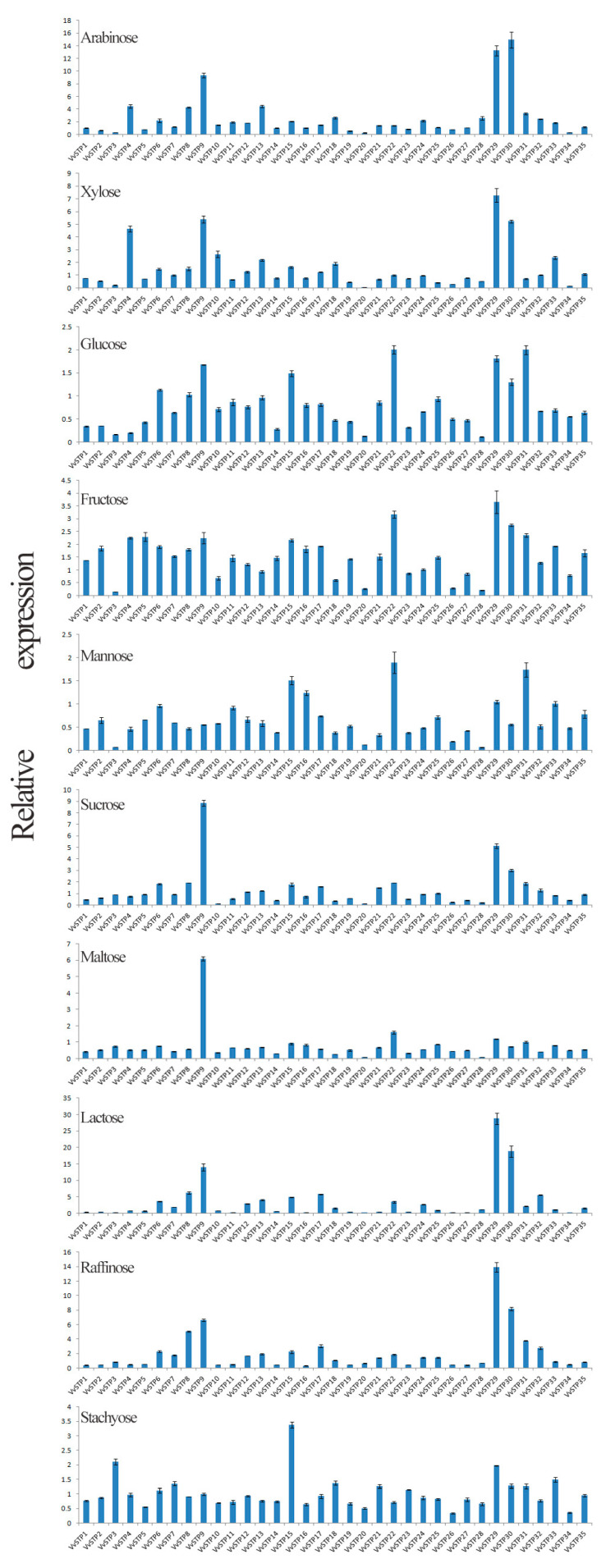
Expression pattern of VvSTPs on different sole carbon sources analyzed through qRT-PCR. The expression of each VvSTP gene in NM medium without sugar was regarded as a reference and the α-tubulin gene was used as an internal control to normalize the data, and relative expression level was calculated through 2^−ΔΔCt^ method. SDs, shown by error bar, were obtained from three biological replicates.

The expression of VvSTPs differed according to the sole carbon source, as shown in Figure 4. Arabinose and fructose generally increase the expression of VvSTPs, while maltose, mannose, and glucose generally decrease the expression of VvSTPs. *VvSTP9* and *VvSTP29* maintained a higher expression level within all VvSTPs and carbon sources, while *VvSTP20* and *VvSTP3* expressed at a lower level. The expression of almost all VvSTPs was reduced when growing on a medium containing sucrose as the sole carbon source, with the exception of *VvSTP22* and *VvSTP29*. The expression of most VvSTPs was also decreased when the sole carbon source of stachyose was used, the most preferred carbon source of *V. virens* [33]. However, the expression of *VvSTP15* and *VvSTP3* increased by over two-fold when stachyose was used. In the presence of glucose, the most important monosaccharide, most VvSTPs were inhibited and only three VvSTPs, *VvSTP22*, *VvSTP31*, and *VvSTP29*, increased more than 1.5 times.

### 2.4. Expression Response of VvSTPs to H_2_O_2_ Treatment

**Figure 5:**
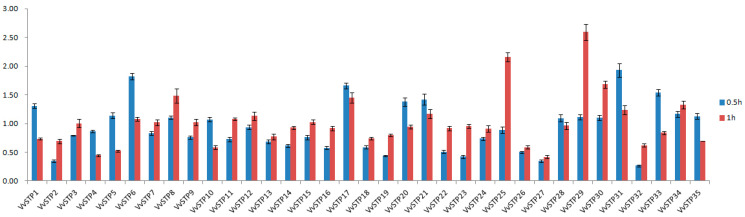
Expression response of *VvSTP*s to H_2_O_2_ through analysis of relative expression with qRT-PCR after treatment of 0.5 and 1 h. The expression of each *VvSTP* gene at 0 h was regarded as a reference and the α-tubulin gene was used as an internal control to normalize the data, and relative expression level was calculated through 2^−ΔΔCt^ method. SDs shown by error bar were obtained from three biological replicates.

During invasion, *V. virens* had to confront reactive oxygen species (ROS), such as superoxide and H_2_O_2_, produced by rice’s oxidative burst, a common plant defense mechanism against pathogen infection. Through qRT-PCR, we measured the relative expression of VvSTPs after treatment with H_2_O_2_ for 0.5 and 1 h, respectively. After 0.5 h of treatment with H_2_O_2_, most VvSTPs did not clearly respond except *VvSTP2*, *VvSTP19*, *VvSTP23*, *VvSTP27*, and *VvSTP32*, whose expression decreased to less than 0.5-fold, while expression of *VvSTP6*, *VvSTP17*, *VvSTP31*, and *VvSTP33* increased more than 1.5-fold. Upon treatment with H_2_O_2_ for one hour, only expression of *VvSTP4* and VvSTP27 decreased to less than 0.5-fold. In contrast, expression of *VvSTP25*, *VvSTP29*, and *VvSTP30* increased more than 1.5-fold, while the other VvSTPs remained relatively stable. According to the combined data from treatments of 0.5 and 1 h, H_2_O_2_ negatively regulated expression of *VvSTP27*, *VvSTP32*, and *VvSTP2*, but positively regulated expression of *VvSTP29*, *VvSTP31*, *VvSTP17*, and *VvSTP25* (Figure 5).

### 2.5. Expression Pattern of VvSTPs after Inoculation on Resistant and Susceptible Varieties

**Figure 6:**
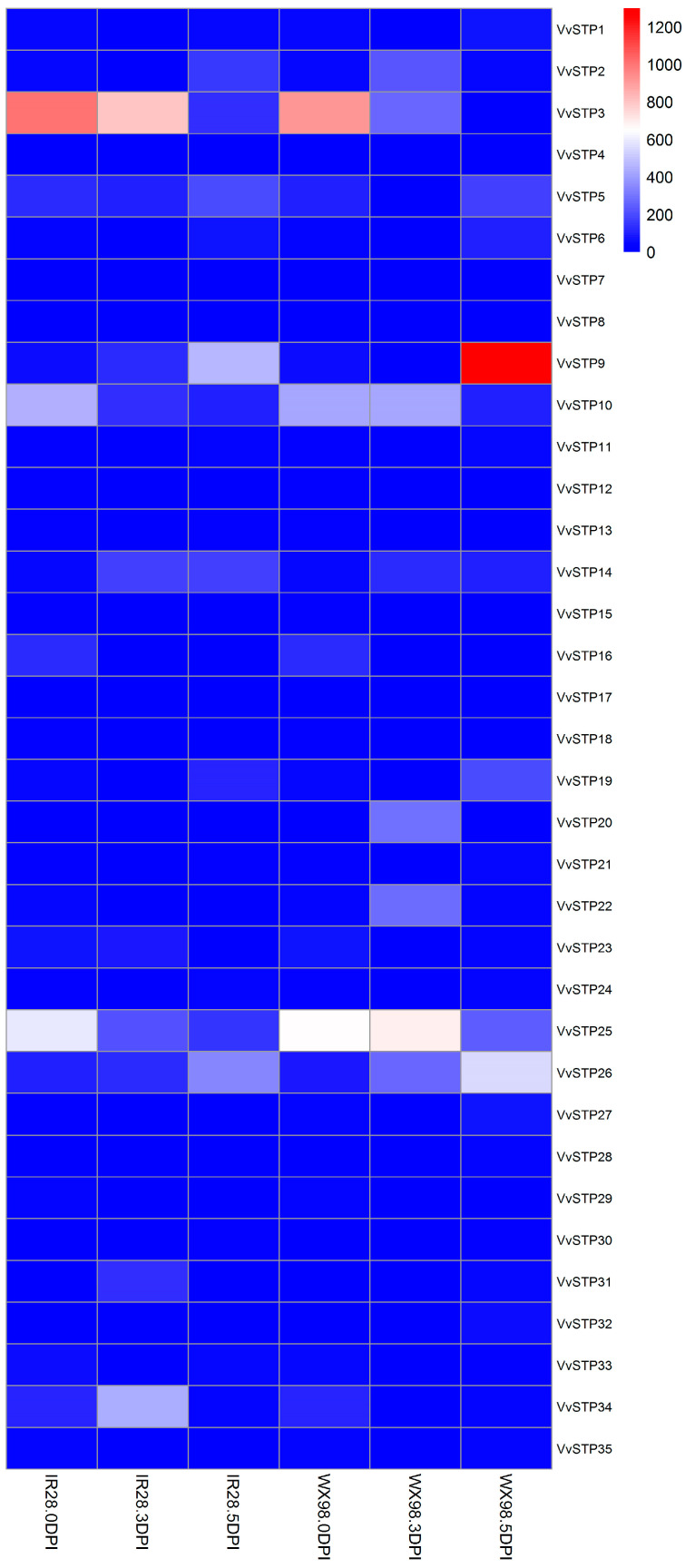
Expression pattern of VvSTPs after inoculation of a resistant variety and a susceptible variety (IR28 and WX98) with *Villosiclava virens*. The heatmap was constructed based on the expression level of each RPKM value of VvSTP genes from RNA-Seq data. Colors from blue to red in boxes indicate expression levels from highest to lowest, respectively. DPI, days post inoculation.

RNA-seq was used to compare the expression pattern of VvSTPs after inoculation on resistant and susceptible varieties. Figure 6 shows that expression patterns of VvSTPs can be divided into five groups based on changes in the trend. Group 1, including *VvSTP* 5, 6, 9, 11, 14, 19, 24, 26, and 30, exhibited a higher expression level 5 DPI in both resistant and susceptible varieties. Group 2, including *VvSTP* 3, 10, 12, 15, 16, 20, 23, 25, 29, and 34, showed a decreased expression level 5 DPI in both resistant and susceptible varieties. *VvSTP*2, *VvSTP*13, and *VvSTP*33 belonged to group 3, in which the expression level on IR28 increased while on WX98, it decreased or remained unchanged compared to the control. In addition, the expression level on IR28 remained the same while on WX98, it declined 5 DPI. On the contrary, *VvSTP*1, 18, 21, 22, 27, 28, 31, 32, and 35 belong to group 4, in which the expression level on IR28 dropped while on WX98, it rose or was unchanged. In addition, the expression level on IR28 remained the same while that on WX98 increased 5 DPI. The rest of *VvSTP*s, including *VvSTP* 4, 7, 8, 17, and 20, were classified into group 5, which did not obviously respond to the inoculation. It is only considered that expression of a gene has been changed when the difference exceeds a 10 FPKM value and 1.5-fold.

### 2.6. Phenotype of V. virens Conferred by Deletion of VvSTP Genes

**Figure 7:**
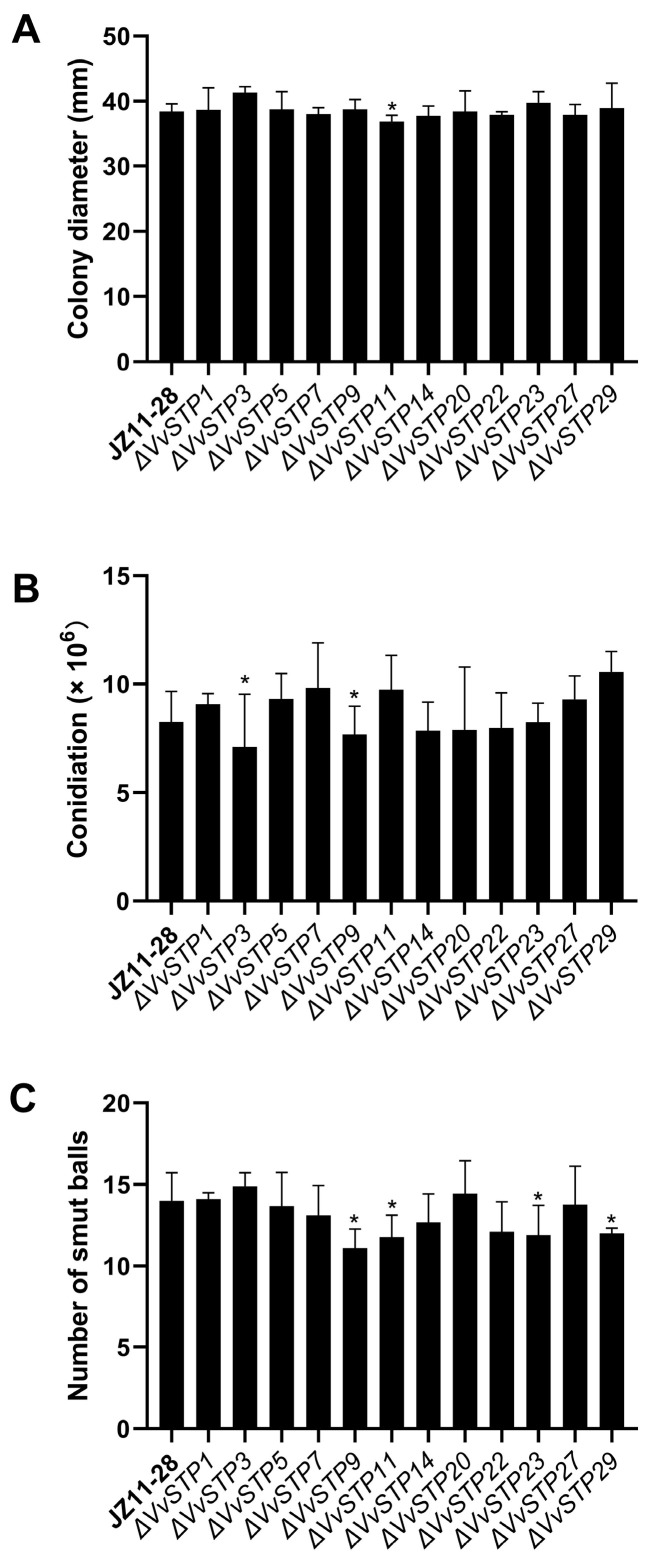
Phenotype assays of VvSTP gene deletion mutants. (**A**) Mycelium growth; (**B**) conidiation; (**C**) pathogenicity. Error bars represent the standard deviation, and asterisks represent a significant difference (*p* < 0.05).

To investigate the role of VvSTP genes in *V. virens* mycelial growth, we incubated the wild strain and VvSTP deletion mutants on PSA plates. VvSTP gene deletion mutants showed similar mycelia growing compared with the wild strain, resulting in similar colony diameters (Figure 7A), indicating the gene had no effect on mycelia growth. Only Δ*VvSTP11* demonstrated a significantly lower growth rate compared with the wild strain, but the distance between them is less than two millimeters. A similar trend was observed for conidiation (Figure 7B). According to our findings, most of the VvSTP gene deletion mutants produced conidia in amounts similar to those produced by the wild strain. There were only two gene deletion mutants that showed significantly lower production than the wild strain. These were Δ*VvSTP3* and Δ*VvSTP9*. The results of these studies indicated that most of the VvSTP genes could not directly affect the vegetative growth of *V. virens*.

Compared with vegetative growth, VvSTP gene deletion mutants showed more distant changes in infection efficiency (Figure 7C). Δ*VvSTP9*, Δ*VvSTP11*, Δ*VvSTP23*, and Δ*VvSTP29* showed a significant decline in pathogenesis, while other mutants did not show a noticeable change in pathogenesis. As a result of these results, it has been demonstrated that VvSTPs influence the pathogenesis of *V. virens* in different ways, and that some VvSTPs play an important role in the pathogenicity of the pathogen.

## 3. Discussion

In this study, 35 VvSTPs were identified from *V. virens* and named from *VvSTP1* to *VvSTP35*. A bioinformatic analysis was used to detect and analyze transmembrane domains, gene structures, and conserved motifs of the VvSTPs. Furthermore, a phylogenetic analysis within the gene family indicated that *VvSTP1* and *VvSTP27* were distantly related to other VvSTPs and to each other. At the fourth node, most VvSTPs were clustered into one group except *VvSTP1*, *VvSTP8*, *VvSTP27*, *VvSTP29*, *VvSTP33*, *VvSTP34*, and *VvSTP35*. In a phylogenetic relationship analysis with other species, most VvSTPs interlaced with STPs from two other filamentous fungi. However, most VvSTPs are more closely related to those of *S. cerevisiae* compared to STPs from two other filamentous fungi. During the pathogenic process, the expression of VvSTPs responded differently to each sole carbon source and to H_2_O_2_, suggesting their roles played in interaction with rice and their functions in pathogenesis. In order to determine their role in the pathogenicity of *V. virens*, several representative VvSTPs were knocked out through genetic recombination. It was found that knock-out mutants of VvSTPs exhibited little difference in mycelia growth and conidiation, which indicates that one gene in this family cannot affect vegetative growth of *V. virens*. However, these mutants have a different effect on infection efficiency, suggesting that VvSTPs play an important role in virens pathogenicity. The findings of our study may contribute to further explanation of the role played by VvSTPs in the pathogenic mechanisms of *V. virens*.

Considering that only 35 VvSTPs were identified in the genome of *V. virens*, we examined the STPs of nine filamentous fungi for comparison, namely *Aspergillus fumigatus*, *Claviceps purpurea*, *Cordyceps militaris*, *Fusarium graminearum*, *Metarhizium acridum*, *M. anisopliae*, *M. robertsii*, *Sclerotinia sclerotiniorum*, and *Magnaporthe oryzae* (Table 2). Almost all other fungi in the study had higher numbers of STP, and the only one with low numbers of STP was *C. purpurea* instead of *M. acridum* and *M. anisopliae*, which had a closer relationship to *V. viren* according to previous studies [32]. The most important rice pathogen, *M. oryzae*, had 122 STPs, nearly four times more than *V. virens*, while two other necrotrophic plant pathogens, *F. graminearum* and *A. fumigatus*, also displayed larger STP numbers, showing 116 and 89, respectively [10,34,35,36,37]. In recent years, there have been more and more indications that *V. virens* is more likely to acquire nutrients from its host in a biotrophic or semibiotrophic manner [20,29,38,39]. It is therefore likely that *V. virens* does not require as many STPs as these necrotrophic plant pathogens due to their nutrient-obtaining mechanisms. We speculate that the similar STP number between *C. purpurea* and *V. virens* may arise from the fact that both pathogens infected crop panicles, which contain the highest concentration of sugars in the plant [40].

It is necessary for STPs to localize on the membrane in the subcellular region in order to perform their function in sugar exchange across membranes, as evidenced by more and more studies [41,42,43,44]. All VvSTPs were predicted to contain 12 transmembrane domains and be located on plasma membranes, suggesting that all VvSTPs are membrane proteins. A majority of VvSTPs had PI values greater than 7, consistent with MeSTPs identified in cassava [44], but *VvSTP24*’s PI was as low as 5.13, which is lower than other identified STPs, suggesting its amino acid composition may differ from other STPs previously identified.

Normally, more introns in eukaryotic genes indicate more complex regulation mechanisms and involvement in more complex biological processes due to the fact that introns may also play an important role in regulating gene expression [45,46]. The number of introns in VvSTPs varied significantly from plants, such as Arabidopsis [47], grapes [48], pears [49], and cassava [50]. The number of introns in *VvSTP16* was higher than that found in other identified STPs from both plants and fungi, suggesting that VvSTPs may be regulated differently and in a more complex manner. It was reported that MFS transporters in plants have a common structure composed of 12 transmembrane domains (TMD1-TMD12), and any STP sequence with less than 10 TMD is not going to be functional. We detected 12 TMDs in all VvSTPs, which indicates a certain degree of similarity between *V. virens* and plants.

A phylogenetic analysis of the VvSTP gene family revealed that *VvSTP1* and *VvSTP27* were separated from other VvSTPs. The length of AA sequences of *VvSTP1* and *VvSTP27* did not differ from other VvSTPs. *VvSTP1* has 11 motifs, which is the most in all VvSTPs. Both *VvSTP1* and *VvSTP27* have as high as five introns, indicating a higher level of evolutionary activity. Our future research will focus on these two genes and attempt to explain the effect of intron variation and N-domains on VvSTPs.

Compared to animals and plants, fewer STP gene families have been systemically investigated in fungi. To analyze phylogenetic relationships with other fungal STP families, we selected *S. cerevisiae*, *N. crassa*, and *U. maydis* as references. It is notable to find that *VvSTP8* and *VvSTP35* showed a closer relationship with STPs from *N. crassa*, such as NCU09321, than with the majority of VvSTPs. It is known that NCU09321 is a sucrose transporter from *N. crassa* [51,52]. There is no other detailed illustration of this gene available. The STPs of *N. crassa* and *U. maydis* were intertwined, indicating a closer phylogenetic relationship and a more distant relationship with *V. virens*. According to comparative genomic evolution, *N. crassa* and *V. virens*, both ascomycetes, show a significantly closer evolutionary relationship than *U. maydis*, a basidiomycete fungus [32]. Considering the enormous genetic differences between *S. cerevisiae*, a yeast, and the other three filamentous fungi, it is not surprising that all STPs of *S. cerevisiae* were placed in a separate clade. In the gene family of the VvSTPs, *VvSTP12* and *VvSTP23* showed a closer phylogenetic relationship to UM02374 (*UmSrt1*), the first fungal STP that has been extensively studied and proven to be essential in the pathogenesis of *U. maydis* [7,53]. Consequently, our lab has knocked out *VvSTP23*, which is likely to be necessary for infection, based on our preliminary research.

The oxidative burst of rice, which uses ROS as the primary weapon, is a defense line that *V. virens* should be prepared to counter from the very beginning of infection. Therefore, we examined the expression of VvSTPs in *V. virens* exposed to H_2_O_2_ treatment at different times. It is thought that the majority of VvSTPs maintained a relatively stable expression level because they are located downstream of the gene regulation pathway and are unable to respond as quickly as upstream genes, such as transcription factors [54]. *VvSTP4*, 27, 25, 29, and 30 showed a relatively violent reaction to H_2_O_2_ treatment and are considered to be likely pathogenic genes.

There were differences in the expression of VvSTPs in response to different sole carbon sources. The expression of VvSTPs is reduced in most sugars, except for arabinose and fructose. It is important to note that panicle, the infection region, is rich in sucrose, which is the most common sugar in rice. For this reason, we were more interested in the expression pattern of VvSTPs in sucrose, in anticipation of discovering some VvSTPs associated with pathogenesis. When the other VvSTPs are depressed, *VvSTP22* and *VvSTP29* exhibit a different expression pattern, which logically raises more concerns. This is only on the basis of culturing in vitro, which is always different from a natural infection; for instance, the expression of *UmSrt1*, a sucrose transporter, was not significantly increased by sucrose in axenic conditions, but was increased after infection^7^. The expression of *VvSTP29* was radically increased in all sugars with fold increases greater than 28, whereas the expression of *VvSTP29* rapidly decreased after inoculation on both resistant and susceptible varieties, IR28 (from 28.37 to 0) and WX98 (from 36.75 to 9.37), according to RNA-sequencing data. In a recent study, stachyose was reported to be the most preferred carbon source of *V. virens* [33], whereas expression of most VvSTPs was depressed when it was used as the sole carbon, which may indicate substrate inhibition of VvSTPs. Most VvSTPs were also inhibited when glucose, the most important monosaccharide, was the only carbon source.

RNA-seq was used to analyze the expression pattern of VvSTPs after inoculation on resistant and susceptible varieties to find more direct evidence for their role in infection processes. In *V. virens*, 5 DPI is a critical period in the disease development, when mycelia massively infect stamen anthers, almost indicating that the infection has been successful. Due to the fact that *V. virens* was unable or only slightly capable of infecting the resistant variety 5 DPI, half of the VvSTPs expressed in IR28 5 DPI could not be detected. In contrast, most of the VvSTPs in WX98 were detected as a result of successful infection. Inoculation results in a variety of expression patterns. The sequence of *VvSTP12* and *VvSTP23* was more similar to *UmSrt1*, but their expression showed significant decline following inoculation, in contrast with *UmSrt1*’s increasing expression following infection^7^. Interestingly, expression of *VvSTP29* could rapidly respond to H_2_O_2_ treatment, sole carbon sugars, and inoculation, but plunged after inoculation, suggesting that it might not be necessary in infection compared with an in vitro culture. It was found that *VvSTP9* expression could be induced by sugar in an axenic condition and kept stable by H_2_O_2_, and was elevated after infection, which made *VvSTP9* the most likely candidate VvSTP involved in pathogenesis, and it is destined to be extensively researched in the future.

## 4. Material and Methods

### 4.1. Identification and Sequence Analysis of Sugar Transporter Genes of V. virens

HEMMER [55] was used to search for STP genes in a published database of *V. virens* protein downloaded from GenBank (accession number: JHTR00000000) [32] with a Pfam [56] HMM (Hidden Markov Model) profile for the Sugar_tr domain (PF00083) at a threshold of an E-value < 0.01. The resulting protein sequences were further submitted into CD-search (http://www.ncbi.nlm.nih.gov/Structure/cdd/wrpsb.cgi accessed on 21 June 2019) to find their containing domains with an E-value threshold of 0.01 against the defaulted database of CDD v3.16-50369 PSSMs [57], and only those sequences with Sugar_tr domains were confirmed as VvSTP candidate genes for a further analysis. Based on their GenBank accession numbers, the verified VvSTP candidate genes are numbered from *VvSTP1* to *VvSTP35*. Compute pI/Mw software (https://web.expasy.org/compute_pi/ (accessed on 22 June 2019)) was used to calculate the theoretical pI (isoelectric point) and Mw (molecular weight) of candidate VvSTPs [58]. VvSTP transmembrane domains and subcellular localization were predicted separately by DeepTMHMM v. 1.0.24 (https://dtu.biolib.com/DeepTMHMM accessed on 18 December 2023) [59] and WoLF PSORT (https://wolfpsort.hgc.jp/ accessed on 22 June 2019) [60].

### 4.2. Gene Structure and Conserved Motifs

Intron and extron features were determined based on genome annotation in GenBank (accession number: JHTR00000000) [32], and displayed using Gene Structure Display Server (GSDS) 2.0 (http://gsds.gao-lab.org/ accessed on 18 December 2023) [61]. GSDS created a picture of gene features, and the result of the phylogenetic analysis of the whole VvSTP gene family was added to the picture to enhance its display. Based on the published protocol, conserved motifs were searched using MEME Suite Programs Version 5.5.5 (https://meme-suite.org/meme/tools/meme accessed on 18 December 2023) with the following settings: maximum number of motifs, 12; motif width, 6 to 100; motif site distribution, zoops [62]. The motif pattern was redrawn using TBtools version 2.031 based on the results generated by the MEME Suite Programs, and a phylogenetic analysis of the entire VvSTP gene family was combined to achieve a more detailed representation [63].

### 4.3. Gene Duplication Analysis and Phylogenetic Analysis of VvSTPs

An all-versus-all blast analysis was performed by searching the whole protein sequence of *V. virens* against a database created with the same sequence set with an e-value of 1 × 10^−5^ by the localized blastp program. The blast output was submitted to the MCScanX program to identify VvSTP gene duplications according to the software manual with defaulted parameters [64].

To better understand the phylogenetic relationships within the VvSTP gene family and the relationships between VvSTPs and STPS gene families of other species, phylogenetic analyses were conducted within the VvSTP gene family and with STP gene families of other fungi. As indicated by published papers, STP gene families from *Saccharomyces cerevisiae* [65], *Neurospora crassa* [51], and *U. maydis* [7] were used as reference genes for the phylogenetic analysis. The STP sequences were aligned using the MUSCLE program using the UPGMA cluster method for the phylogenetic analysis [66]. The result alignment was trimmed with TrimAl version 1.2 with the option to use a heuristic method to decide which was the best automated method [67]. Phylogenetic analyses were performed using iq-tree2 for Linux [68]. In order to perform a high-quality phylogenetic analysis, ModelFinder was used to calculate the best-fit model [69], and then the maximum-likelihood (ML) method was used to estimate two phylogenetic trees by applying the best-fit model with ultra-fast bootstrap approximation for 1000 replicates. The phylogenetic tree of VvSTPs’ family was created with LG+F+R4, the best-fit model chosen according to BIC. The ML analysis of STP genes of *V. virens* and 3 other reference fungi was performed with LG+F+R6, the best testing model also chosen according to BIC. The resulting trees were displayed and illustrated by iTOL [70].

### 4.4. Strain and Variety, Inoculation Experiments, and RNA-Seq

The *V. virens* strain JZ-11-28 and rice varieties WX98 and Yongyou 15 (susceptible to false smut) and IR28 (resistant to false smut) were used for inoculation experiments, and the inoculation process followed previously published procedures [54]. Mycelial plugs punched from a 14~20-day-old colony were transferred into a liquid potato sucrose broth (PSB). After 5~7 days shaken at 180 rpm in a 27 °C culture chamber, aggregated mycelia were cut into small mycelia fractions by a blender; then, the conidia density was adjusted to 10^6^ per ml with PSB. Conidia suspension was inoculated into panicles from the middle to upper portion with a syringe when the rice plants were in the late booting stage (3–5 days before heading). Immediately following inoculation, the plants were transported to a chamber and kept at 27 °C with a relative humidity of over 90% until sampling was performed.

The inoculated spikelet samples were collected 0 and 5 days post inoculation (DPI), when mycelia had massively infected stamen anthers, and the glumes of these spikelet samples were peeled and the florets were retained for RNA extraction in order to analyze the expression pattern of VvSTP genes during pathogenesis. Total RNA was extracted with a Trizol reagent (Invitrogen, Carlsbad, CA, USA) according to the manufacturer’s instructions, and sequenced with a sequence platform (Hiseq2000). The quality of raw data was checked using FastQC 0.11.3, and adaper-removed and quality filtered by Trimmomatic 0.32 with a parameter of LEADING: 3, TRAILING: 3, SLIDINGWINDOW: 4: 15, MINLEN: 50. With the obtained clean reads, the fragments-per-kilobase-per-million-fragments-mapped (FPKM) values of the *VvSTP* genes were calculated after HTSeq counting using the genome of *V. virens* UV-8b (http://www.ncbi.nlm.nih.gov/nuccore/JHTR00000000.1 accessed on 11 September 2018) as the reference. A heatmap was generated using the TBtools version 2.031 based on the calculated FPKM values of VvSTP genes collected at different times after the inoculation of samples.

### 4.5. Quantitative Real-Time PCR Analysis

In PSB media, V. virens strain JZ-11-28 was grown until substantial conidia were formed and mycelia were removed using gauze. We added the filtrate to new PSB media to achieve a conidial density of 10^8^ per mL.

The conidial suspension was shaken at 180 rpm and 27 °C for 48 h to make conidia germinate and mycelia grow, and then centrifuged at 3500× *g* for 10 min, and the supernatant was discarded. The remaining pellet was suspended with an NM medium (0.3% KNO_3_, 6.25% salt solution) [7], and then centrifuged and suspended 3 times to remove residual sugar from the PSA medium and un-germinated conidia. Mycelia suspensions of *V. virens* strain JZ-11-28 in the NM medium were supplemented with various sugars, such as arabinose, xylose, glucose, fructose, mannose, sucrose, maltose, lactose, raffinose, and stachyose, at a concentration of 1% (*w*/*v*). To analyze the expression of VvSTPs on different sole carbon sources, the mycelia were centrifuged after being shaken at 27 °C and 180 rpm for 24 h.

To investigate the expression pattern of VvSTPs under H_2_O_2_ treatment, 6-day-old mycelia from PSB were collected through filtration with gauze and rinsed three times in sterile water. Mycelia were collected for RNA extraction after being treated with 2 mM H_2_O_2_ for 0, 0.5, and 1 h.

For RT-qPCR, total RNA was extracted from *V. virens* mycelia using TaKaRa MiniBEST Plant RNA Extraction Kit (TaKaRa, Dalian, China). DNA removal and first-strand cDNA synthesis were conducted using PrimeScript™ RT reagent Kit including gDNA Eraser (TaKaRa Biotechnology (Dalian) Co., Ltd., Dalian, China) with RT Primer Mix, a mixture of 6 random mers and oligo dT primers, as recommended by the manual. Gene-specific primers for each *VvSTP* were designed by Primer3 [71]. This RT-qPCR mixture was prepared with 10 μL of 2 × SYBR Premix Ex Taq II (TaKaRa, Dalian, China), 0.2 μL of each primer, and 1 μL of the RT product. RT-qPCR was performed on the ABI7500 platform using the following parameters: 50 °C for 2 min; 95 °C for 10 min; 40 cycles of 95 °C for 15 s; and 60 °C for 1 min. Three replicates were used for all expression analyses. The α-tubulin gene was used as an internal control to normalize the data, and the relative expression level was calculated through the 2^−ΔΔCt^ method [72].

### 4.6. Generation of VvSTP Gene Deletion Mutants and Phenotype Assays

The VvSTP genes were knocked out using a protocol described in one of our previous publications [73]. A double-joint PCR method was used to construct the DNA fragments for the genetic recombinant [74], and the detail was also described in our paper [73]. By using the standard PEG-mediated method [75], we directly transformed the wild-type strain JZ-11-28, replacing the target VvSTP genes with the hygromycin resistance gene to produce VvSTP gene deletion mutants.

Phenotype assays were performed to investigate the functions of VvSTPs. For the mycelium growth test, a plug of fungi 5 mm in diameter was placed on a PSA plate and cultured at 28 °C for 10 days. For conidiation, the strains were cultured in the PSB medium at 28 °C for 7 days with 160 rpm shaking. In the next step, conidia concentrations were determined using a hemocsytometer after the cultured mixture was filtered. As described in a published paper [75], rice was inoculated with V. virens to test its pathogenicity. As described in Section 2.4, the conidia suspension was prepared and adjusted to 5 × 10^6^ per ml with PSB. A syringe was used to inject approximately 2 mL of the mixed suspension of conidia and hyphals in PSB into a single rice panicle from the middle to upper portion during the late booting stage (3–5 days before heading). The inoculated plants were maintained in a greenhouse at 27 °C and 90–100% relative humidity (RH) for seven days, followed by placing them at 27 °C and 80% RH until rice false smut symptoms were observed. Smut balls were measured 21 days after inoculation in order to observe symptoms and determine the frequency of occurrence. Each of the experiments in this section was conducted in at least three independent biological experiments with at least three replicates per experiment.

## Figures and Tables

**Table 1:** Information of the predicted *STP* genes in *Villosiclava virens*.

Gene ID	Access Number	Chromosome	Gene Size	AA Length	PI	MW (kDa)	TMD	Subcellular Localization
*VvSTP1*	XP_043001649	7	2199	732	7.27	80.93	12	Plasma membrane
*VvSTP2*	XP_043001702	7	2016	671	7.56	74.92	12	Plasma membrane
*VvSTP3*	XP_042999314	4	1659	552	8.95	61.50	12	Plasma membrane
*VvSTP4*	GAO15224	3	1882	541	9.02	59.64	12	Plasma membrane
*VvSTP5*	XP_042994690	1	2019	672	6.87	75.55	12	Plasma membrane
*VvSTP6*	XP_042994673	1	1899	632	8.76	70.19	12	Plasma membrane
*VvSTP7*	XP_042994564	1	1761	586	7.01	62.84	12	Plasma membrane
*VvSTP8*	XP_042996797	3	1584	527	9.22	57.39	12	Plasma membrane
*VvSTP9*	XP_042998161	4	1656	551	6.98	60.17	12	Plasma membrane
*VvSTP10*	XP_042994317	1	1599	532	6.9	58.27	12	Plasma membrane
*VvSTP11*	XP_042995010	1	1722	573	8.17	61.47	12	Plasma membrane
*VvSTP12*	XP_042995051	1	1611	536	9.04	57.44	12	Plasma membrane
*VvSTP13*	XP_043001089	6	1563	520	9.21	54.58	12	Plasma membrane
*VvSTP14*	XP_042998431	1	2142	713	9.09	77.91	12	Plasma membrane
*VvSTP15*	XP_042993682	1	1560	519	6.44	56.69	12	Plasma membrane
*VvSTP16*	XP_042994804	1	2334	777	9.34	86.56	12	Plasma membrane
*VvSTP17*	XP_042999475	5	1497	498	6.84	54.12	12	Plasma membrane
*VvSTP18*	XP_042993975	1	1677	558	9.14	60.71	12	Plasma membrane
*VvSTP19*	XP_042994240	1	1674	557	5.69	60.75	12	Plasma membrane
*VvSTP20*	XP_043001123	7	1494	497	8.92	54.59	12	Plasma membrane
*VvSTP21*	XP_042995111	1	1506	501	8.31	53.81	12	Plasma membrane
*VvSTP22*	XP_043000182	5	1584	527	8.6	57.75	12	Plasma membrane
*VvSTP23*	XP_043000657	6	1581	526	6.97	55.69	12	Plasma membrane
*VvSTP24*	XP_042998299	4	1656	551	5.13	60.48	12	Plasma membrane
*VvSTP25*	XP_042993458	1	1890	629	8.26	70.23	12	Plasma membrane
*VvSTP26*	XP_042998199	4	1656	551	9	59.96	12	Plasma membrane
*VvSTP27*	XP_042993861	1	1653	550	8.55	60.95	12	Plasma membrane
*VvSTP28*	XP_042993876	1	1752	583	5.64	64.89	12	Plasma membrane
*VvSTP29*	XP_043001720	7	1533	510	9.18	54.75	12	Plasma membrane
*VvSTP30*	XP_043000610	6	1626	541	9.26	59.40	12	Plasma membrane
*VvSTP31*	XP_042995206	2	1605	534	7.07	59.58	12	Plasma membrane
*VvSTP32*	XP_042995203	2	1608	535	8.55	58.13	12	Plasma membrane
*VvSTP33*	XP_042999770	5	1560	519	6.27	56.75	12	Plasma membrane
*VvSTP34*	XP_042998602	4	1674	557	7.97	61.46	12	Plasma membrane
*VvSTP35*	XP_042999744	5	1548	515	8.69	57.26	12	Plasma membrane

**Table 2:** Number of *STP*s in *V. virens* and other filamentous fungi.

Species	Number
*Villosiclava virens*	35
*Aspergillus fumigatus*	89
*Claviceps purpurea*	33
*Cordyceps militaris*	57
*Fusarium graminearum*	116
*Metarhizium acridum*	89
*Metarhizium anisopliae*	62
*Metarhizium robertsii*	59
*Sclerotinia sclerotiorum*	61
*Magnaporthe oryzae*	122

## Data Availability

The data presented in this study are available on request from the corresponding author.
